# In Memoriam of Iván Lásló Bonta (1922–2007)

**DOI:** 10.1155/2007/38283

**Published:** 2007-03-19

**Authors:** Freek J. Zijlstra

**Affiliations:** Department of Anesthesiology, Erasmus MC, P.O. Box 2040, 3000 CA, Rotterdam, The Netherlands

Professor Emeritus Iván Lásló Bonta, an internationally renowned pharmacologist, died in Rome, Italy, on 6 February 2007, at age 84, on the verge of boarding an airplane to Israel. His death is mourned by his family, many colleagues, friends, and former students.

Iván was born on 6 October 1922 in Budapest, Hungary. Due to antisemitic laws, after his grammar school, he was not permitted to pursue medical studies. He survived the 2nd World War in a forced labour camp in Poland. Thereafter, he studied medical science in Budapest and graduated in 1950. He became a doctoral student and obtained his Ph.D. degree in experimental pharmacology, at a research institute of the Hungarian Army. In 1947, he married Vera. In the early 1950s, his son Janos and daughter Zsoka were born. In 1956, following the Russian suppression of the Hungarians, the family decided to leave Hungary and moved to the Netherlands, where he became Head of Pharmacology Research at the drug company Organon.

After successful research activities at Organon, in 1969 he was appointed Professor and Chairman at the Department of Pharmacology of the Medical School of the Erasmus University Rotterdam. In 1992, he retired from the chair, quite reluctantly at the age of 70 years. Prior to his retirement, in 1991 he founded the international scientific journal *Mediators of Inflammation* (Rapid Communications of Oxford Ltd.), of which the first bimonthly issue was published in February 1992. In 1997 he resigned as Editor-in-Chief.

After the passing away of his wife Vera in 1996, after a restless period, he searched for new challenges and happiness, which he found in 1997 in Israel, where he met Lea Egózi, whom he married and lived with in Nethanya.

His main line of his research was inflammation and immunopharmacology. During his career, he has been (co)author of more than 225 scientific papers.


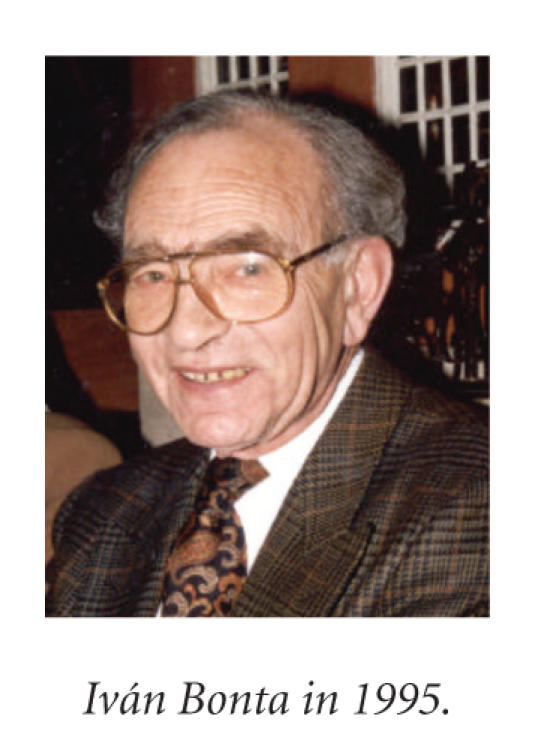
During his time at Organon, he already investigated the effects of serotonin and anti-inflammatory agents in the rat paw oedema [1, 2]. In the Erasmus University Rotterdam, he initiated research with isolated tissue preparations [3, 4], performed by his first two Ph.D. students, of whom another twelve would follow through the years. At that time, in the early 1970s, prostaglandins were discovered. Their role in platelets and the rat paw model of inflammation were the subject of a number of investigations [5–8]. After the discovery of leukotrienes, their biological activity [9] and role in inflammatory cells were investigated [10–12]. As a next step, the involvement of stimulated macrophages and their substances released were investigated as tools against tumour growth [13–17]. However, not only animal and human cells were subjected to immunopharmacological research activities. In the 1980s, the organ bath technique for isolated human tissue was used to study responses of bioactive substances during inflammation [18, 19].

Living in Israel, he initiated research on images of diseases in archaic societies. He was fascinated by pre-Columbian sculpted statuettes which resemble the phenomenology of diseases. Specimens of his impressive private collection have been shown as illustrations in a remarkable book written by him, entitled “*Diseased Ancestors: Essays and Stories around Medical Archaeology.*” In the last years, he was impressed by alternative medicine [20] and associations between schizophrenia, anaesthesia, and near-death experiences [21]. As a result, he wrote a paperback on this subject in Dutch, which has just recently been published.

Iván had a remarkable and also a fruitful life. Through his authorship, research, teaching, and editor's work, he contributed substantially to the immunopharmacology. We will all remember him as a person that liked to teach, to be heard, with a vast knowledge and many stories to tell, someone who enjoyed being in the center of life and attention. In general, he was a charismatic, charming, erudite, and enjoyable person, with a lot of very typical humour.

He is survived by his present wife Lea, his son Janos and his wife Gladys, his daughter Zsoka and her husband Edgar, and his three grandchildren, Peter, Ilana, and Anne.

On Sunday 11 February 2007, we buried him in the Jewish cemetery in Rotterdam, next to his first wife Vera as he wished it to be.

*Freek J. Zijlstra* *Freek J. Zijlstra*
